# Association of DNA methylation with hypertension and blood pressure: a 7-year longitudinal study from KORA F4/FF4

**DOI:** 10.1186/s12916-026-04672-8

**Published:** 2026-02-04

**Authors:** Liye Lai, Angelina Shin Yee Jong, Thomas Delerue, Jiesheng Lin, Barbara Thorand, Margit Heier, Holger Prokisch, Aiman Farzeen, Juliane Winkelmann, Elisabeth Thiering, Christian Gieger, Annette Peters, Melanie Waldenberger

**Affiliations:** 1 Research Unit Molecular Epidemiology, Helmholtz Zentrum München, German Research Center for Environmental Health (GmbH), Neuherberg, Germany; 2 Institute of Epidemiology, Helmholtz Zentrum München, German Research Center for Environmental Health (GmbH), Neuherberg, Germany; 3 Institute for Medical Information Processing, Biometry, and Epidemiology (IBE), Pettenkofer School of Public Health, Faculty of Medicine, Ludwig Maximilians University, Munich, Germany; 4 School of Public Health and Emergency Management, School of Medicine, Southern University of Science and Technology, Shenzhen, China; 5 KORA Study Centre, University Hospital of Augsburg, Augsburg, Germany; 6 Institute of Neurogenomics, Computational Health Center, Helmholtz Zentrum München, Neuherberg, Germany; 7 Institute of Human Genetics, School of Medicine, Technical University Munich, Munich, Germany; 8 Cluster for Systems Neurology (SyNergy), Munich, Germany; 9 Chair of Neurogenetics, Technische Universität München, Munich, Germany; 10 Department of Pediatrics, Dr. Von Hauner Children’s Hospital, LMU University Hospital, Munich, Germany; 11 German Center for Diabetes Research (DZD), Neuherberg, Germany; 12 German Centre for Cardiovascular Research (DZHK), Partner Site Munich Heart Alliance, Munich, Germany

**Keywords:** DNA methylation, Hypertension, Blood pressure, Methylation trajectory, Gene expression

## Abstract

**Background:**

Hypertension (HTN) has been linked to changes in DNA methylation. However, longitudinal epigenome-wide analyses are still limited.

**Methods:**

We analyzed data from the KORA F4 and FF4 studies, conducted approximately 7 years apart. The dataset included 2614 participants, each with DNA methylation measured at least once. Leucocyte DNA methylation was profiled using the Illumina 450 k and EPIC arrays. Linear mixed-effects models were employed to identify associations between methylation sites and HTN status, systolic (SBP) and diastolic blood pressure (DBP). Interaction terms with follow-up time captured longitudinal methylation trajectories. We further examined CpG sites related to reversed, persistent, or progressive HTN and assessed their correlations with gene expression.

**Results:**

One CpG site was associated with SBP and four with DBP, all representing novel loci, including *RILP* (cg08625564) and *SVIL* (cg15298791). Differential annual methylation changes were observed for 2, 23, and 12 CpG sites by HTN status, SBP, and DBP, respectively, highlighting genes such as *RHPN2*, *CLDND1*, *ZNF69*, and *FKBP1B*. Twenty CpG sites were associated with persistent HTN, including *PLCB2* and *MPPE1*. In whole blood, 22 significant CpG–transcript pairs were detected, involving 14 CpG sites and 19 gene transcripts.

**Conclusions:**

This longitudinal epigenome-wide study identified novel CpG sites associated with blood pressure and persistent HTN. We observed differential DNA methylation trajectories over time linked to HTN, SBP, and DBP, with several changes correlating with gene expression, suggesting functional relevance. These findings underscore the dynamic role of DNA methylation in blood pressure regulation and provide new insights into epigenetic mechanisms of HTN.

**Graphical Abstract:**

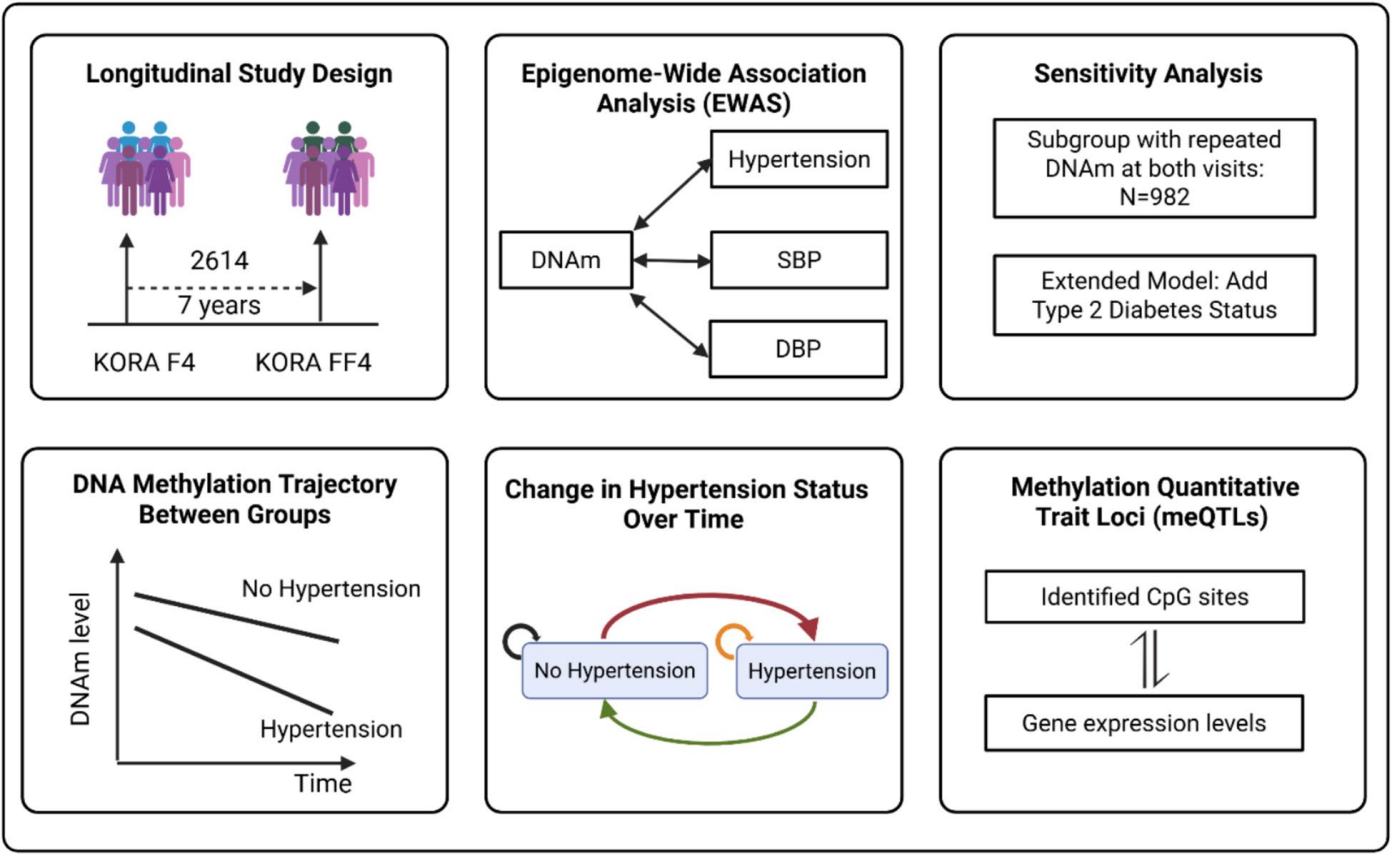

**Supplementary Information:**

The online version contains supplementary material available at 10.1186/s12916-026-04672-8.

## Novelty and relevance

What Is New?

1. Conducted large-scale longitudinal analyses with repeated DNAm measurements.
2. Identified differential methylation trajectories associated with HTN status over time.

What Is Relevant?

1. Demonstrates longitudinal links between DNA methylation, HTN, and BP.

Clinical Implications

1. Emphasizes the dynamic role of DNA methylation in the regulation of BP.
2. Offers new insight into epigenetic mechanisms of HTN.

## Background

Hypertension (HTN) is a major global health challenge, contributing to pathological cardiac hypertrophy and cardiovascular morbidity and mortality. According to the World Health Organization (WHO), it affects over a billion people worldwide, approximately 1 in 4 men and 1 in 5 women. Despite extensive research, the mechanisms driving HTN remain incompletely understood. DNA methylation has emerged as critical mediators linking genetic, environmental, and lifestyle factors to blood pressure regulation and cardiovascular disease [1].

Cross-sectional epigenome-wide association studies (EWAS) have identified numerous CpG sites associated with HTN and related traits, with meta-analyses further refining these associations. For example, a two-stage EWAS meta-analysis of 17,010 multi-ancestry individuals identified 13 replicated CpG sites explaining 1.4–2.0% of blood pressure variation beyond known genetic variants, with several showing causal links to gene expression through mendelian randomization [2, 3]. Complementing this, another meta-analysis replicated 34 CpG sites, six of which were associated with gene expression, with twin-based modeling suggesting that their associations with blood pressure were primarily driven by shared environmental rather than genetic factors [4]. Prenatal and perinatal environments also shape epigenetic regulation of blood pressure, as hypertensive disorders of pregnancy are linked to altered DNA methylation in newborns, while maternal blood pressure correlates with placental methylation both directly and through changes in placental cell-type composition, with potential implications for offspring development [5, 6].

Cross-sectional designs, however, cannot clarify the temporal relationship between methylation changes and disease development. Given the dynamic nature of the epigenome, longitudinal studies are essential. For example, in 1072 Chinese twins, cross-lagged analyses showed that certain CpG sites predict future blood pressure changes, with some bidirectional effects, while methylation risk scores were associated with HTN progression, underscoring the value of longitudinal epigenetic analyses for clarifying temporal relationships [7]. Similarly, in 380 African Americans followed for approximately 5 years, generalized estimating equation models identified 22 CpG sites with significant longitudinal methylation changes [8], further supporting the importance of longitudinal designs. Moreover, longitudinal methylation studies in specific populations provide additional mechanistic insights. For instance, in obese patients undergoing Roux-en-Y gastric bypass, genome-wide methylation profiling before and 6 months after surgery identified novel CpG sites linked to essential HTN, emphasizing the dynamic nature of methylation in response to physiological and therapeutic interventions [9].

Taken together, although extensive evidence links DNA methylation to blood pressure and HTN, most studies rely on cross-sectional or single-time-point measurements, limiting understanding of temporal dynamics. Our study leverages two-time-point longitudinal methylation data to examine how changes in DNA methylation relate to blood pressure trajectories, enabling the identification of predictive epigenetic markers and providing mechanistic insights into HTN development that prior studies could not capture.

## Methods

### Study population

This study used data from the Cooperative Health Research in the Region of Augsburg (KORA) F4 (2006–2008) and FF4 (2013–2014) studies, both follow-up studies of the KORA S4 study (1999–2001). Detailed information on the KORA cohort design, measurement, and data collection has been previously described [10]. In total, 3596 observations from 2614 participants in KORA F4 (1724) and FF4 (1872), with methylation data at least once across two visits, were included in the analysis. Of these participants, 982 participants from 1964 observations had methylation patterns measured at both time points. Detailed information about the inclusion of study participants can be found in Additional file 1: Text S1.

### Measures of epigenome-wide DNA methylation and gene expression

In the KORA F4 study, genome-wide DNA methylation in whole blood was analyzed using the Illumina 450 K Infinium Methylation BeadChip (Illumina Inc., San Diego, CA, USA). For the KORA FF4 study, the Infinium MethylationEPIC BeadChip (Illumina Inc., San Diego, CA, USA) was used. DNA methylation was quantified on a scale of 0 to 1, with 1 signifying 100% methylation. We followed the general outline of the CPACOR preprocessing for quality control by using the minfi2 package. A total of 374,054 CpG sites were left for the analysis, and detailed information about the quality control step and inclusion of CpG sites can be found in Additional file 1: Texts S2–S3. The proportions of white blood cell types (CD8T, CD4T, natural killer (NK) cells, B lymphocytes, monocytes and granulocytes) were estimated using the Reinius reference-based houseman algorithm implemented in the minfi package. The algorithm is based on methylation values obtained from purified cell types in whole blood. These proportions were then utilized as covariates in the model to mitigate cell type confounding. The KORA F4 and FF4 datasets each included 470 and 448 non-negative control probes from the methylation arrays, respectively, with 430 probes overlapping. To address technical effects during the experiment, we conducted principal component analysis (PCA) on the overlapping probes. The resulting principal components (PCs) are believed to capture technical variability, and the first five control probe PCs, which accounted for 70% of the variance, were included as covariates in the model to eliminate technical biases.

Gene expression data were available only from the KORA FF4 study. Of the 1872 individuals with DNA methylation data in KORA FF4, 1543 also had RNA-seq data. This subset includes participants from the group of 982 individuals with repeated methylation measurements across KORA F4 and FF4. RNA-seq generation and processing are detailed in Additional file 1: Text S4. After quality control, expression data from these 1543 participants, comprising 10,671 genes, were retained for downstream analyses.

### Measures of HTN status

HTN was defined based on the WHO definition, with systolic blood pressure (SBP) ≥ 140 mm Hg and/or diastolic blood pressure (DBP) ≥ 90 mm Hg, or reported use of antihypertensive medications given that participants were aware of having hypertension. Blood pressure was measured using an OMRON HEM-705CP device after participants had been seated for at least 5 min. Three measurements were taken at 3-min intervals, and the average of the second and third measurements was used for analysis. Information on antihypertensive medications was classified by the guidelines of the German Hypertension Society.

### Statistical analysis

#### Epigenome-wide association studies

Focusing on the KORA F4 (*n* = 1724) and FF4 (*n* = 1872) participants, we applied linear mixed-effects models with random participant-specific intercepts to examine the associations between HTN status (exposure) and DNA methylation (outcome). The primary model was adjusted for follow-up time (defined as the interval between two questionnaire surveys: 0 for the baseline survey, and the time difference from baseline to subsequent follow-up survey), age at baseline (years), sex (male, female), body mass index (BMI, kg/m^2^), smoking status (never, former, current), estimated cell types (monocytes, B Cells, CD4 T cells, CD8 T cells, and NK cells), and technical effects. We used the false discovery rate (FDR) (Benjamini–Hochberg method) to account for multiple testing. An association was considered statistically significant at a *p_*FDR value < 0.05. The same linear mixed-effects model was used to investigate the association between DNA methylation and two continuous outcomes—SBP and DBP—with additional adjustment for antihypertensive medication use.

#### Sensitivity analysis

We conducted two sensitivity analyses to evaluate the robustness of our findings. First, in the full cohort of KORA F4 (*n* = 1724) and FF4 (*n* = 1872) participants, we expanded our primary model by additionally adjusting for diabetes status. Second, we performed a restricted analysis using the same primary model but including only the 982 participants who had repeated measures of both DNA methylation and HTN-related traits at both time points.

#### DNA methylation trajectories over time

To investigate differential DNA methylation trajectories between traits, an interaction term between HTN, SBP, or DBP and follow-up time was added to the primary model, in the full cohort of KORA F4 (*n* = 1724) and FF4 (*n* = 1872) participants. For HTN, this interaction captures the difference in the rate of annual methylation change between individuals with and without HTN. For SBP and DBP, it represents the difference in the rate of annual methylation change associated with a one-unit (1 mmHg) increase in blood pressure. The resulting trajectories exhibited two patterns: converging, where methylation differences between groups decreased over time, and diverging, where group differences increased over time.

#### Association between DNA methylation and changing HTN status over time

Since this study involves longitudinal data, individuals could change their HTN status between baseline and follow-up. We focused on a subset of participants with repeated DNA methylation measurements (*N* = 982) at both time points and excluded 443 individuals who reported use of antihypertensive medication. To explore disease progression over time, we examined transitions between normotension and HTN and categorized the remaining 539 individuals into four groups: (1) stable normotension: 27 individuals with normotension at both time points; (2) reversion: 25 individuals who transitioned from HTN to normotension; (3) stable HTN: 456 individuals with HTN at both time points; (4) progression: 31 individuals who progressed from normotension to HTN. Linear mixed-effects models were constructed with time-varying HTN status as the exposure and methylation as the outcome, adjusting for the same covariates as in the primary analysis, such as follow-up time, age at baseline, sex, BMI, smoking status, estimated cell types, and technical effects.

#### Association between DNA methylation and gene expression

To investigate the relationship between DNA methylation and gene expression and to enhance the functional annotation of our findings, we performed a cis-methylation quantitative trait loci (cis-mQTL) analysis. This analysis was restricted to the 1543 KORA FF4 participants who had both DNA methylation and RNA-seq data available and focused on the significant CpG sites identified in this study. We tested for associations between each CpG site and all gene expression probes located within a 500-kb window. Using the MatrixEQTL package (version 2.3), we constructed general linear models with methylation levels as the exposure and gene expression levels as the outcome, adjusting for age, sex, measured white blood cell proportions (neutrophils, monocytes, basophils, eosinophils), and technical variation. Significance was assessed using FDR correction for multiple testing. Detailed methods are provided in Additional file 1: Text S5.

## Results

### Characteristics of the study population

The study population included 1724 participants in KORA F4 and 1872 in KORA FF4, with HTN prevalence at 45.7% in F4 and 35.9% in FF4 as described in Table 1. Across both cohorts, hypertensive individuals were consistently older, with average ages around 64 years, compared to mid-to-late 50 s in normotensive participants. Although the overall mean age in FF4 was slightly lower than in F4, this is largely explained by the younger age of the reference (normotensive) group in FF4, which lowers the overall average despite similar or slightly higher ages among hypertensives. Comparison of study population characteristics between the overall cohort and participants with repeated measurements is provided in Additional file 1: Table S1. Men were more likely to be hypertensive, and BMI was significantly higher in hypertensives across both time points. There was a trend toward lower current smoking rates among hypertensive individuals, likely reflecting lifestyle changes after diagnosis. Blood pressure readings were predictably higher in the hypertensive group, but systolic and diastolic pressures were slightly better controlled in FF4, suggesting improved clinical management over time. The prevalence of type 2 diabetes was markedly higher among hypertensives in both cohorts, reflecting the strong metabolic link between these conditions.

**Table 1:** Characteristics of the study population

Characteristics	KORA F4			KORA FF4		
	All *N* = 1724	Ref *N* = 936	HTN *N* = 788	All *N* = 1872	Ref *N* = 1200	HTN *N* = 672
Age (years)	61.0 (8.89)	58.2 (8.42)	64.2 (8.30)	58.6 (11.6)	55.1 (10.8)	64.8 (10.3)
Male (%)	842 (48.8)	397 (42.6)	443 (56.2)	891 (47.6)	536 (44.7)	354 (52.7)
BMI (kg/m^2^)	28.1 (4.78)	26.7 (4.11)	29.8 (4.98)	27.8 (5.12)	26.7 (4.51)	29.8 (5.54)
Smoking						
Never smoker (%)	721 (41.8)	387 (41.5)	334 (42.4)	768 (41.0)	496 (41.4)	272 (40.5)
Former smoker (%)	753 (43.7)	381 (40.9)	371 (47.1)	794 (42.4)	471 (39.3)	322 (47.9)
Current smoker (%)	248 (14.4)	164 (17.6)	83 (10.5)	310 (16.6)	231 (19.3)	78 (11.6)
SBP (mmHg)	125.0 (18.7)	117.0 (12.9)	134.0 (19.9)	119.0 (17.2)	113.0 (12.6)	128.0 (19.9)
DBP (mmHg)	76.0 (10.0)	73.5 (7.6)	79.1 (11.5)	73.4 (9.5)	71.8 (7.6)	76.1 (11.7)
Diabetes mellitus (%)	158 (9.16)	32 (3.43)	126 (16.0)	162 (8.65)	38 (3.17)	124 (18.4)
HDL-cholesterol (mmol/l)	1.46 (0.38)	1.53 (0.39)	1.38 (0.35)	1.69 (0.49)	1.75 (0.49)	1.59 (0.47)
Triglycerides (mmol/l)	1.52 (1.01)	1.34 (0.86)	1.72 (1.14)	1.40 (0.85)	1.27 (0.72)	1.64 (1.00)
LDL-cholesterol (mmol/l)	3.62 (0.91)	3.66 (0.92)	3.57 (0.90)	3.49 (0.89)	3.49 (0.85)	3.50 (0.95)
Cholesterol (mmol/l)	5.73 (1.01)	5.80 (1.01)	5.65 (1.00)	5.60 (1.00)	5.61 (0.94)	5.58 (1.08)

Data are mean (SD) for continuous variables and n (%) for categorical variables

### Association between DNA methylation and HTN/SBP/DBP

**Fig. 1:**
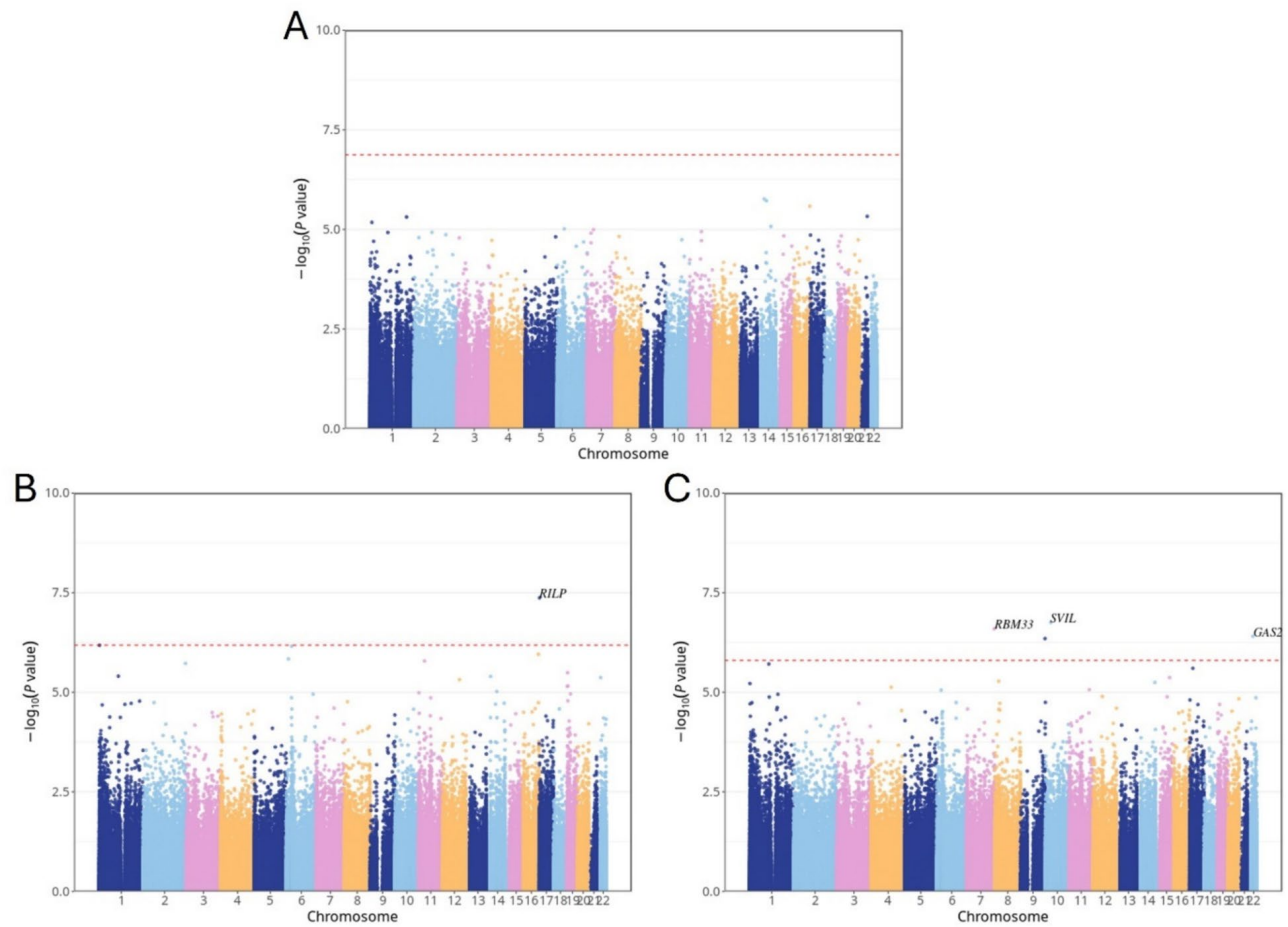
Manhattan plot illustrating EWAS results associated with HTN, SBP, and DBP. The *x*-axis indicates the chromosome location, and the *y*-axis represents the − log10 (*p*-value). The Benjamini–Hochberg (FDR) threshold (*p_*FDR < 0.05) is indicated by a red dashed line

An EWAS was conducted to examine the relationship between DNA methylation, HTN, SBP, and DBP, using linear mixed-effects models with individual-specific random intercepts. The analysis included 374,054 CpG sites measured in individuals with partly repeated DNA methylation data. No CpG sites reached statistical significance for HTN status after multiple testing correction. However, one CpG site was significantly associated with SBP, and four CpG sites were significantly associated with DBP at a FDR threshold of 0.05. All five significant CpG sites demonstrated positive effect sizes, indicating hypermethylation in relation to higher SBP or DBP values. The most significant CpG site associated with SBP was cg08625564, annotated to the *RILP* (Rab-interacting lysosomal protein) gene (FDR = 0.0159; effect size = 1.42e − 4), located on chromosome 17. For DBP, four CpG sites surpassed the FDR significance threshold: cg15298791 (*SVIL*: Supervillin), cg13639901 (*RBM33*: RNA Binding Motif Protein 33), cg01259126 (*GAS2L1*: Growth Arrest Specific 2 Like 1), and cg14559176 (unannotated). These CpG sites were located on chromosomes 10, 7, 22, and 9, respectively. In total, five CpG sites corresponding to four unique genes were identified, all representing novel blood pressure–associated CpG sites. The Manhattan plot (Fig. 1) visualizes the genome-wide distribution of associations for HTN, SBP, and DBP, highlighting the significant CpG sites. Table 2 and Additional file 2: Tables S1–S3 provide a detailed summary of these loci.

**Table 2:** Summary of significant CpG sites associated with SBP/DBP

Probe ID	BETA	P_VAL	FDR	CHR	MAPINFO	UCSC_RefGene
**SBP**						
cg08625564	1.42e − 4	4.25e − 08	1.59e − 02	17	1,553,453	*RILP*
**DBP**						
cg15298791	1.48e − 4	1.77e − 07	4.20e − 02	10	29,827,523	*SVIL*
cg13639901	3.47e − 4	2.55e − 07	4.20e − 02	7	155,556,590	*RBM33*
cg01259126	2.06e − 4	3.93e − 07	4.20e − 02	22	29,705,157	*GAS2L1*
cg14559176	1.98e − 4	4.49e − 07	4.20e − 02	9	138,872,899	*#*

*Probe ID *unique identifier from the Illumina CG database, *BETA* the estimate from the linear mixed model, *FDR* Benjamini–Hochberg corrected *p *value, *CHR *chromosome, *UCSC_RefGene *target gene name from the UCSC database (# indicates no annotated gene), *MAPINFO *chromosomal coordinates of the CpG (Build 37)

### Sensitivity analysis

As a sensitivity analysis, we extended the main model by further adjusting for diabetes status. Among the 374,054 CpG sites examined, one site for SBP and four sites for DBP remained statistically significant and directionally consistent with the main analysis results. Additionally, we conducted a subset analysis using 982 individuals with repeated DNA methylation measurements at two time points. In this subset, no CpG sites reached FDR significance for associations with HTN, SBP, or DBP. However, the correlation of effect estimates between the full cohort (*N* = 2614) and the repeated-measures subset (*N* = 982) remained strong, with Pearson correlation coefficients of *r* = 0.80, 0.77, and 0.78 for HTN, SBP, and DBP, respectively. The Manhattan plot can be found in Additional file 1: Fig. S1, and the detailed summary of these loci can be found in Additional file 2: Tables S4–S9.

### Time-dependent associations between blood pressure traits and DNA methylation trajectories

To assess whether DNA methylation trajectories differ by HTN status and blood pressure levels over time, we applied linear mixed-effects models including interaction terms between follow-up time and HTN (or SBP/DBP). In this model, β1 represents the rate of methylation change over time in the reference group (non-hypertensive), β2 captures the interaction effect (the difference in slope between groups), and β3 (β1 + β2) reflects the trajectory slope in the hypertensive or higher BP group. We identified 2, 23, and 12 CpG sites with significant time-by-HTN, time-by-SBP, and time-by-DBP interaction effects, respectively. All 2 time-by-HTN interaction CpG sites and the top 6 time-by-SBP/DBP interaction CpG sites are shown in Table 3; the rest are provided in Additional file 2: Tables S10–S12.

**Table 3:** Summary of significant CpG sites with different methylation rates change over time

Probe ID	BETA_1	BETA_2	BETA_3	P_VAL	FDR	CHR	MAPINFO	UCSC_RefGene
**HTN**								
cg12165551	− 1.52e − 2	0.15e − 2	− 1.37e − 2	3.19e − 08	1.19e − 02	11	8,385,712	*#*
cg01580888	− 0.32e − 2	− 0.15e − 2	− 0.47e − 2	2.22e − 07	4.16e − 02	19	33,556,060	*RHPN2*
**SBP**								
cg10739556	− 0.76e − 2	0.57e − 4	− 0.75e − 2	1.64e − 15	6.12e − 10	7	27,192,056	*#*
cg25438440	− 0.77e − 2	0.38e − 4	− 0.77e − 2	4.36e − 15	7.44e − 10	3	98,241,168	*CLDND1*
cg03608000	− 0.61e − 2	0.31e − 4	− 0.61e − 2	5.96e − 15	7.44e − 10	19	11,998,623	*ZNF69*
cg11866851	− 0.96e − 2	0.34e − 4	− 0.96e − 2	6.78e − 11	6.34e − 06	5	31,532,348	*RNASEN*
cg03687700	− 2.72e − 2	1.15e − 4	− 2.71e − 2	1.57e − 10	1.17e − 05	2	24,271,844	*FKBP1B*
cg25697152	− 1.59e − 2	0.54e − 4	− 1.58e − 2	1.95e − 10	1.22e − 05	12	3,067,777	*TEAD4*
**DBP**								
cg10739556	− 0.70e − 2	0.86e − 4	− 0.69e − 2	8.12e − 12	3.04e − 06	7	27,192,056	*#*
cg03608000	− 0.58e − 2	0.47e − 4	− 0.58e − 2	2.38e − 11	4.45e − 06	19	11,998,623	*ZNF69*
cg25438440	− 0.72e − 2	0.54e − 4	− 0.71e − 2	1.95e − 10	2.43e − 05	3	98,241,168	*CLDND1*
cg16383222	− 1.34e − 2	0.69e − 4	− 1.33e − 2	4.71e − 10	4.40e − 05	8	145,688,536	*CYHR1*
cg08615596	− 1.36e − 2	0.60e − 4	− 1.35e − 2	8.73e − 10	6.53e − 05	12	68,758,840	*#*
cg03687700	− 2.64e − 2	1.78e − 4	− 2.62e − 2	1.81e − 08	1.13e − 03	2	24,271,844	*FKBP1B*

*Probe ID*, unique identifier from the Illumina CG database. BETA_1 represents the estimated methylation change rate per year over follow-up time for individuals without HTN or with SBP/DBP equal to zero (i.e., the reference group). BETA_2 is the estimate of the interaction between HTN status (or SBP/DBP levels) and follow-up time, indicating how the methylation change rate differs between hypertensive (or higher blood pressure) individuals and the reference group. BETA_3 is the combined methylation change rate per year for individuals with HTN or higher blood pressure, calculated by adding BETA_1 and BETA_2; this reflects the total slope of methylation change over time in the hypertensive/high blood pressure group. P_VAL: for the interaction effect, specifically for BETA_2. *FDR*, Benjamini-Hochberg corrected *p* value. *UCSC_RefGene*, target gene name from the UCSC database

**Fig. 2:**
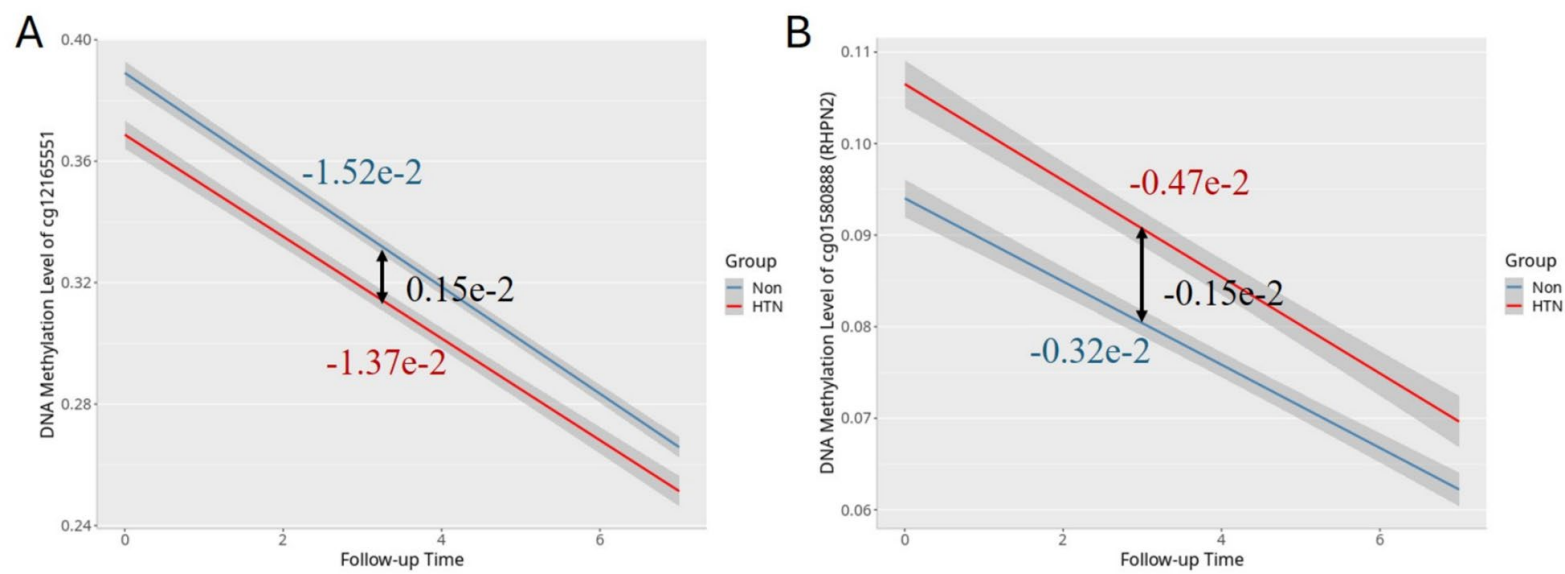
Line plots illustrating the rate of methylation change over time for the reference (blue line) and HTN (red line) groups. **A** cg12165551; **B** cg01580888 (*RHPN2*)

For HTN, two CpG sites—cg12165551 and cg01580888 (*RHPN2*: Rhophilin Rho GTPase Binding Protein 2)—showed significant interactions. Both sites exhibited converging trajectory patterns, as illustrated in Fig. 2, where the methylation level differences between the hypertensive and non-hypertensive groups diminished over time. This convergence depends on the combined interpretation of the interaction term direction (β₂) and the baseline methylation status of the two groups (BETA: can be found in Additional file 2: Table S10). At cg12165551, a positive β₂ (0.15e − 2) indicated a slower methylation decline in the hypertensive group; combined with the hypomethylated status of the hypertensive group at baseline, this narrowed the inter-group gap. Meanwhile, at cg01580888, a negative β₂ (− 0.15e − 2) indicated a faster methylation decline in the hypertensive group; coupled with the hypermethylated status of the hypertensive group at baseline, this similarly reduced the inter-group difference. As illustrated in the Fig. 2, the red (HTN) and blue (non- HTN) lines represent β3 and β1 slopes, respectively, with their convergence or divergence reflecting the direction and magnitude of β2.

Among the SBP-associated CpG sites, cg03608000 (*ZNF69*: Zinc Finger Protein 69), cg03687700 (*FKBP1B*: FKBP Prolyl Isomerase 1B), and cg25438440 (*CLDND1*: Claudin Domain Containing 1) showed the strongest effects. All these three CpG sites were also significantly associated with DBP, indicating consistent associations across both blood pressure traits.

### DNA methylation with HTN trajectory: reversion, persistence, and progression

**Fig. 3:**
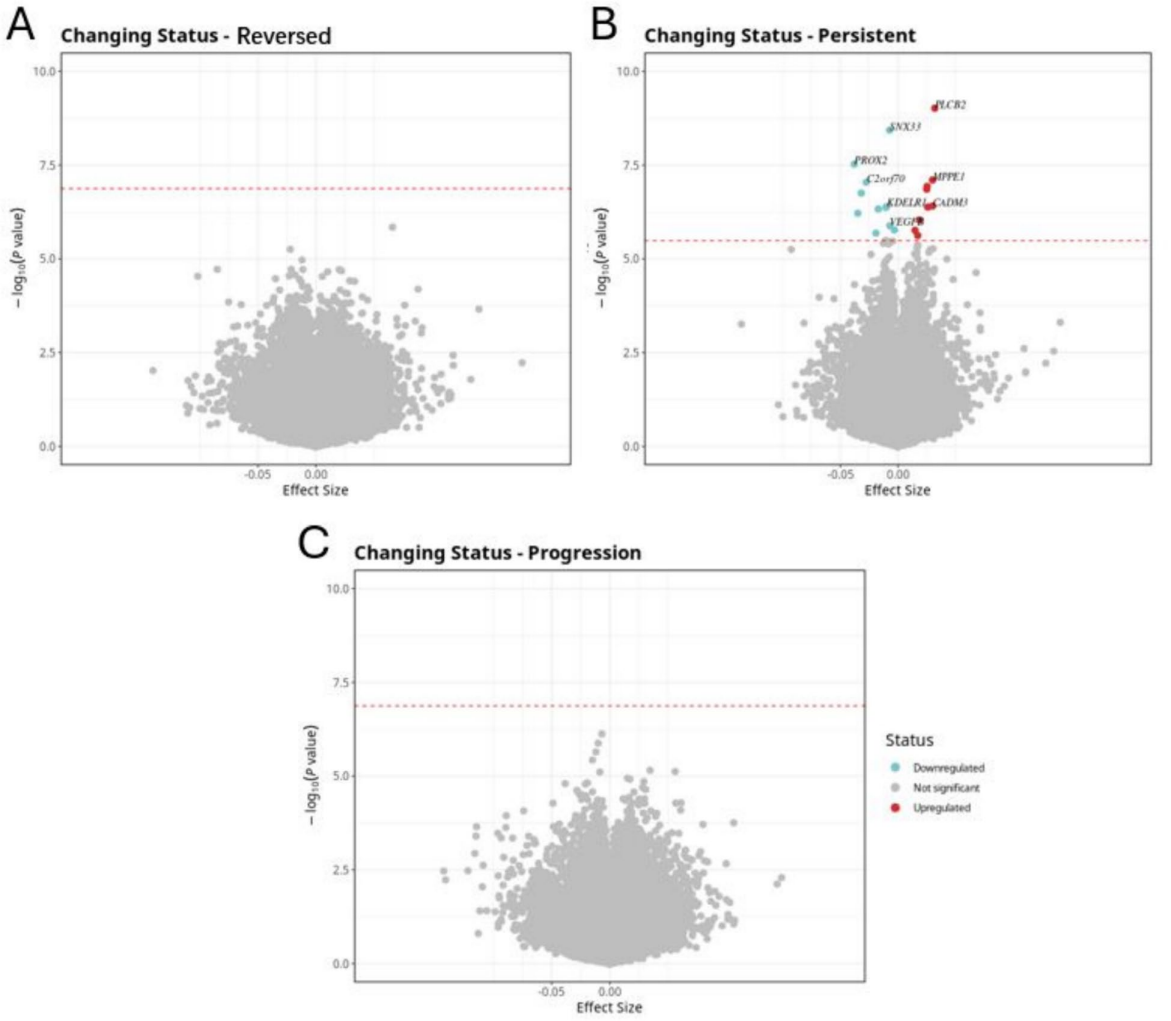
Volcano plots illustrating the association between DNA methylation and changing HTN status over time. The *x*-axis indicates the effect size, and the *y*-axis represents the − log10 (*p*-value). The Benjamini–Hochberg (FDR) threshold (*p_*FDR < 0.05) is indicated by a blue dashed line. **A** Volcano plot for the reversed HTN. **B** Volcano plot for the persistent HTN. **C** Volcano plot for the progression of HTN

We focused on a subset of participants with repeated DNA methylation data at two time points (*N* = 982). After excluding 443 individuals who reported use of antihypertensive medication, 539 individuals remained for the final analysis. We examined the association between DNA methylation and HTN transition status, categorized into persistent, reversed, and progressed groups. Four distinct blood pressure trajectories were identified. The reference group maintained stable SBP (114.0 mmHg) and DBP (72.7 to 71.8 mmHg). The persistent group showed increased SBP (149.0 to 154.0 mmHg) with stable DBP (91.7 to 90.0 mmHg). The reversed group exhibited decreased SBP (142.0 to 124.0 mmHg) and DBP (85.2 to 74.3 mmHg). The progressed group demonstrated increased SBP (125.0 to 146.0 mmHg) and DBP (77.7 to 83.2 mmHg). A total of 20 CpG sites were significantly associated with persistent HTN (FDR < 0.05), while no significant CpG sites were identified for the reversed or progressed groups. Among the significant sites, cg25033076 annotated to *MPPE1* (Metallophosphoesterase 1, FDR = 6.78 × 10⁻^3^), previously reported in association with blood pressure traits, was also detected in our analysis. The effect sizes (β values) for significant CpG sites ranged from − 0.04 to + 0.03, indicating both hypo- and hypermethylation patterns in individuals with persistent HTN compared to the reference group. The associations are illustrated in a volcano plot (Fig. 3), and the corresponding results are summarized in Additional file 1: Table S2 and Additional file 2: Tables S13–15.

### Association between DNA methylation and gene expression

In the KORA FF4 cohort (*N* = 1543), we investigated CpG–transcript associations in whole blood using RNA-seq data (10,671 transcripts) for a total of 62 CpG sites previously found to be significantly associated with HTN-related phenotypes. These included: 1 site for SBP, 4 for DBP, 2 for HTN interaction, 23 for SBP interaction, 12 for DBP interaction, and 20 for persistent HTN—corresponding to 51 unique CpG sites. Among these, we identified 22 significant CpG–transcript associations, involving 14 unique CpG sites and 19 unique gene transcripts (FDR < 0.05). Of the 22 associations, 5 showed positive directionality (mean effect size: + 0.78) and 17 showed negative directionality (mean effect size: − 1.49). For example, cg25033076, annotated to *MPPE1*, was also associated with transcripts such as *TUBB6* (Tubulin Beta 6 Class V), *AFG3L2* (AFG3 Like Matrix AAA Peptidase Subunit 2), and *CHMP1B* (Charged Multivesicular Body Protein 1B). Similarly, cg03608000, annotated to *ZNF69*, was linked to multiple transcripts including *ZNF439* (Zinc Finger Protein 439), *ZNF44* (Zinc Finger Protein 44), *PRKCSH* (PRKCSH Beta Subunit of Glucosidase II), and *ZNF763* (Zinc Finger Protein 763). Additional file 1: Table S3 and Additional file 2: Table S16 present the significant CpG–transcript associations.

## Discussion

This study employed longitudinal data with repeated measurements to explore the association between DNA methylation and HTN status, as well as blood pressure. A total of 3,596 observations from 2,614 participants, each with DNA methylation measured at least once across two visits, were included in the analysis. We identified one CpG site associated with SBP and four with DBP, all representing novel loci. Additionally, 2, 23, and 12 CpG sites showed differential annual methylation change rates by HTN status, SBP, and DBP, respectively, which exhibited either faster or slower decreasing trends. We also identified 20 CpG sites associated with persistent HTN. Finally, in whole blood, we detected 22 associations between identified significant HTN-related CpG sites and their corresponding gene expression levels.

In our study, the CpG site cg08625564, annotated to the *RILP* gene, was significantly associated with SBP. Although not previously linked to blood pressure in epigenetic studies, *RILP* has been implicated in several pathways relevant to cardiovascular and metabolic health. A recent single-cell transcriptomics study showed that *RILP* expression was downregulated in diabetic hearts following ischemia–reperfusion injury, contributing to impaired autophagic flux and increased myocardial vulnerability, which could be restored by targeting the mTOR/*RILP* pathway, thereby exerting cardioprotective effects [11]. *RILP* also induces cholesterol accumulation in lysosomes by inhibiting endoplasmic reticulum–endo lysosome interactions [12] and mediates macrolipophagy in diabetic cardiomyopathy, where Rab7 activation restored *RILP* expression and improved cardiac function [13]. Additionally, *RILP* affects insulin secretion [14] and acts as a tumor suppressor silenced by hypermethylation in lung cancer [15]. These findings support a potential role for *RILP* methylation in SBP regulation via pathways involving autophagy and lysosomal function.

Four CpG sites—cg15298791 (*SVIL*), cg13639901 (*RBM33*), cg01259126 (*GAS2L1*), and cg14559176—were significantly associated with DBP, all showing positive effect estimates and representing novel loci linked to DBP. A large-scale genome-wide association study involving 5900 hypertrophic cardiomyopathy (HCM) cases and 68,359 controls identified 70 loci associated with HCM, among which rare truncating variants in *SVIL* conferred an approximately tenfold increased risk, establishing *SVIL* as a novel HCM disease gene [16]. Supporting this, exome sequencing reanalysis of 200 HCM patients further highlighted *SVIL* variant carriers presenting with apical and septal HCM, acropathies, and skeletal abnormalities such as severe scoliosis [17]. Mechanistically, loss of *SVIL* has been shown to cause myopathy characterized by myofibrillar disorganization and accumulation of autophagic vacuoles, underscoring its role in maintaining muscle structure and autophagic processes [18]. Taken together, these findings suggest that *SVIL* plays a critical role in cardiovascular and muscular integrity and supports a potential mechanistic link between epigenetic regulation of *SVIL* and vascular function. *RBM33* has been identified as an m6A RNA-binding protein that modulates *ALKBH5* (AlkB Homolog 5, RNA Demethylase) demethylase activity and RNA substrate specificity [19, 20]. Reduced *RBM33* expression has also been linked to moyamoya disease, a cerebrovascular disorder involving arterial occlusion [21], supporting its involvement in vascular function. These findings point to a potential epigenetic mechanism by which *RBM33* may influence blood pressure.

The prevalence of type 2 diabetes was markedly higher among hypertensives in both cohorts, reinforcing the strong metabolic link between these conditions. As a sensitivity analysis, we extended the main model by further adjusting for diabetes status. One site for SBP and four sites for DBP remained statistically significant and directionally consistent with the main analysis results. This finding suggests that the associations with HTN and blood pressure are not solely explained by diabetes. The results remain robust, independent of metabolic comorbidities.

A total of 2, 23, and 12 CpG sites showed differential annual methylation change rates by HTN status, SBP, and DBP, respectively. For HTN, one example is cg01580888 annotated to *RHPN2*. Notably, *CLDND1*, *ZNF69*, and *FKBP1B* were among the blood pressure–related genes identified in both SBP- and DBP-associated methylation dynamics. A functional analysis identified the cancer-associated single-nucleotide variants (SNVs) rs10411210, whose risk allele upregulates *RHPN2* expression and enhances RhoA activation, linking *RHPN2* to cancer-related Rho GTPase signaling [22]. *CLDND1*, a tight junction protein, is upregulated in the cerebellum of stroke-prone hypertensive rats due to reduced microRNA-124, linking it to cerebrovascular disease [23]. *CLDND1* expression is elevated in ischemic stroke patients, especially among current smokers, potentially contributing to their increased stroke risk [24]. Smoking-related alterations in DNA methylation and gene expression, including changes in the *CLDND1* gene, have been associated with cardiometabolic traits [25, 26]. Recent studies have highlighted the role of FK506-binding protein 1B (*FKBP1B*) in cardiovascular function. *FKBP1B* regulates intracellular cardiac calcium levels and modulates ryanodine receptor activity, influencing calcium-dependent nitric oxide (NO) release and promoting vasodilation. This mechanism contributes to reduced blood pressure. Conversely, deletion of *FKBP1B* leads to impaired calcium handling, resulting in cardiac hypertrophy and HTN in mice [27–29]. Additionally, *FKBP1B* variants have been shown to alter spontaneous activity in human induced pluripotent stem cell-derived cardiomyocytes (hiPSC-CMs), suggesting a direct role in modulating cardiac electrophysiological properties [30]. The interaction model showed that DNA methylation at certain CpG sites decreases with age, but declines at a different rate in individuals with high blood pressure or HTN. This suggests that elevated BP alters the natural epigenetic aging process, pointing to disrupted epigenetic remodeling linked to HTN. Consistent with this, methylation-based biological age has been shown to be positively associated with both prevalent and incident hypertension [31]. Furthermore, antihypertensive medication use has been associated with greater methylation age acceleration over time, indicating that methylation age may not fully capture the protective, risk-reducing effects of taking antihypertensive medication [32].

Individuals with persistent HTN show significant differences in methylation at specific CpG sites compared to those who remained normotensive, while those who developed or improved HTN status do not show such differences. Examples include loci annotated to *PLCB2* (phospholipase C beta 2), Ubiquitin-Specific Protease 7 (*USP7*), and *CADM3* (Cell Adhesion Molecule 3). *PLCB2* was identified as a key gene in the calcium signaling pathway linked to immune cell infiltration in obesity-related carotid atherosclerosis. Its expression was upregulated in adipose, aortic tissues, and serum of obesity and carotid atherosclerosis model mice [33]. Early-onset cardiovascular and renal diseases have a strong genetic basis. A previous study conducted exome-wide association analyses in a Japanese population and identified *PLCB2* as significantly associated with susceptibility to early-onset myocardial infarction, HTN, and chronic kidney disease [34]. *USP7*, the first identified deubiquitinating enzyme, participates in a variety of biological processes, such as cell proliferation, DNA damage response, tumorigenesis, and apoptosis. *USP7* expression was elevated in Ang II-induced cardiac hypertrophy and remodeling in mice, as well as in humans with heart failure. Treatment with the *USP7* inhibitor P22077 alleviated cardiac hypertrophy, fibrosis, inflammation, and oxidative stress, suggesting *USP7* as a potential therapeutic target for hypertrophic remodeling and heart failure [35]. *USP7* mediates platelet-derived growth factor-induced pulmonary arterial smooth muscle cells (PASMCs) proliferation [36]. Plasma *CADM3* levels were cross-sectionally associated with LV mass-to-volume ratio in the Multi-Ethnic Study of Atherosclerosis (MESA) cohort, suggesting its potential as a biomarker for early LV remodeling and heart failure risk [37]. In a large epigenome-wide study of 13,433 diverse individuals, *CADM3* was identified as a novel CpG site associated with blood CRP levels, a marker of systemic inflammation, suggesting its epigenetic role in chronic inflammation [38]. Our analysis identified differential DNA methylation associated with persistent HTN, but not with new-onset or resolved HTN. This suggests that sustained high blood pressure may lead to stable and detectable epigenetic changes, potentially reflecting long-term vascular, metabolic, or inflammatory effects. In contrast, transient or new-onset HTN might not cause methylation changes large or stable enough to be observed within the study period. Similarly, improvement in HTN may involve recovery or reversal processes that are not strong enough to produce distinct methylation patterns compared to those without HTN. Overall, these findings indicate that chronic exposure to elevated blood pressure is more likely to drive lasting epigenetic alterations than temporary changes in HTN status.

Previous research focuses on the association between the HTN and gene expression levels directly [39]. Using the mQTL approach, we identified 22 CpG–transcript pairs. Notably, in several cases, the gene transcript associated with methylation at a CpG site differed from the Illumina-annotated gene based on proximity, highlighting alternative CpG–gene relationships and suggesting potential long-range regulatory (cis/trans) effects. For example, cg25033076 is annotated to *MPPE1* based on genomic proximity but was also associated with the expression of *TUBB6* and *AFG3L2*, suggesting a broader regulatory influence within the local gene neighborhood or shared promoter/enhancer regions. While no prior studies have linked *MPPE1* to HTN or blood pressure traits, related genes show relevant associations: *TUBB6* polymorphisms have been linked to ischemic stroke risk and outcomes in the Russian population [40] and identified as a potential hub gene for early diagnosis and immune modulation in non-alcoholic fatty liver disease (NAFLD) [41]; *AFG3L2* has been implicated in genome-wide DNA methylation changes in monozygotic twins discordant for myocardial infarction [42]. Similarly, cg03608000, annotated to *ZNF69*, was negatively associated with multiple transcripts including *ZNF439*, *ZNF44*, *PRKCSH*, and *ZNF763*, reflecting complex regulatory relationships within the *ZNF* gene cluster on chromosome 19. Pathogenic variants in *PRKCSH* are linked to autosomal dominant polycystic liver disease (ADPLD), a rare disorder with a female predominance. Female patients carrying *PRKCSH* mutations and exhibiting rapid liver volume progression are at the highest risk for symptomatic disease and liver-related hospitalization [43–45]. These results highlight that DNA methylation may influence gene expression beyond its nearest annotated gene, potentially acting through shared regulatory domains or chromatin interactions.

Our study has notable strengths. First, we performed a longitudinal analysis over 7 years that incorporated DNA methylation profiles and hypertension status. Second, we integrated gene expression data to support the functional relevance of methylation changes. Finally, we used multiple statistical models to account for potential confounders, increasing the robustness of our results. Several limitations should also be considered. DNA methylation measured in blood may not fully capture tissue-specific patterns. Although we incorporated principal components derived from overlapping non-negative control probes as covariates in all regression models to capture technical variation related to array processing and probe performance, we cannot completely rule out the presence of batch effects. Critically, this study demonstrates a longitudinal association but does not confirm a causal relationship between DNA methylation and the development or progression of hypertension. Additionally, our findings are based on individuals of European ancestry and lack replication in cohorts of diverse ancestry, underscoring the need for future validation across varied populations.

## Conclusions

This longitudinal epigenome-wide association study identified several novel CpG sites associated with blood pressure traits, and associations between DNA methylation levels and persistent HTN status. We further observed differential DNA methylation trajectories over time related to HTN, SBP, and DBP. Notably, several of these methylation changes were associated with gene expression differences, suggesting potential functional relevance. These findings highlight the dynamic nature of DNA methylation in relation to blood pressure regulation and provide new insights into the epigenetic mechanisms of hypertension.

## Supplementary Information

Additional file 1: Text S1. Selection criteria of individuals in KORA F4 and FF4. Text S2. CPACOR Preprocessing Pipeline. Text S3. Selection criteria of CpG sites in KORA F4 and FF4. Text S4. Quality control for KORA FF4 gene expression data. Text S5. DNA methylation and gene expression analysis. Table S1. Characteristics of the study population: overall cohort and participants with repeated measurements. Figure S1. Manhattan plots of sensitivity analysis. Table S2. Summary of significant CpG sites associated with hypertension persistence. Table S3. Significant associated CpGtranscript pairs.

Additional file 2: Table S1. Non-Significant CpG sites associated with HTN. Table S2. Significant CpG sites associated with SBP. Table S3. Significant CpG sites associated with DBP. Table S4. Non-Significant CpG sites associated with HTN from extended model. Table S5. Significant CpG sites associated with SBP from extended model. Table S6. Significant CpG sites associated with DBP from extended model. Table S7. Non-Significant CpG sites associated with HTN from repeated methylation measurements. Table S8. Non-Significant CpG sites associated with SBP from repeated methylation measurements. Table S9. Non-Significant CpG sites associated with DBP from repeated methylation measurements. Table S10. Significant CpG sites associated with interaction between HTN and follow-up time. Table S11. Significant CpG sites associated with interaction between SBP and follow-up time. Table S12. Significant CpG sites associated with interaction between DBP and follow-up time. Table S13. Non-Significant CpG sites associated with reversed HTN. Table S14. Significant CpG sites associated with persistent HTN. Table S15. Non-Significant CpG sites associated with progressed HTN. Table S16. Associated CpG-transcripts pairs.

## Data Availability

The dataset(s) supporting the conclusions of this article is (are) included within the article (and its additional files). The KORA data are available upon request from KORA Project Application Self-Service Tool (https://www.helmholtz-munich.de/en/epi/cohort/kora); data requests can be submitted online and are subject to approval by the KORA Board.

## Abbreviations

*AFG3L2*: AFG3 Like Matrix AAA Peptidase Subunit 2
*ALKBH5*: AlkB Homolog 5, RNA Demethylase
BP: Blood pressure
*CADM3*: Cell Adhesion Molecule 3
*CHMP1B*: Charged Multivesicular Body Protein 1B
*CLDND1*: Claudin Domain Containing 1
DBP: Diastolic blood pressure
EWAS: Epigenome-wide association studies
*FKBP1B*: FKBP Prolyl Isomerase 1B
*GAS2L1*: Growth Arrest Specific 2 Like 1
HTN: Hypertension
KORA: Cooperative Health Research in the Region of Augsburg
*MPPE1*: Metallophosphoesterase 1
PCA: Principal component analysis
*PLCB2*: Phospholipase C Beta 2
*PRKCSH*: PRKCSH Beta Subunit of Glucosidase II
*RBM33*: RNA Binding Motif Protein 33
*RHPN2*: Rhophilin Rho GTPase Binding Protein 2
*RILP*: Rab-interacting lysosomal protein
SBP: Systolic blood pressure
*SVIL*: Supervillin
*TUBB6*: Tubulin Beta 6 Class V
*USP7*: Ubiquitin-Specific Protease 7
*ZNF44*: Zinc Finger Protein 44
*ZNF69*: Zinc Finger Protein 69
*ZNF439*: Zinc Finger Protein 439
*ZNF763*: Zinc Finger Protein 763
