# Supplementary material for: Association of DNA methylation with hypertension and blood pressure: a 7-year longitudinal study from KORA F4/FF4
Source: BMC Med. 2026 Feb 4;24:125. doi: 10.1186/s12916-026-04672-8 (PMC12930669; doi:10.1186/s12916-026-04672-8)
Supplement: Supplementary file 1 — Additional file 1: Text S1. Selection criteria of individuals in KORA F4 and FF4. Text S2. CPACOR Preprocessing Pipeline. Text S3. Selection criteria of CpG sites in KORA F4 and FF4. Text S4. Quality control for KORA FF4 gene expression data. Text S5. DNA methylation and gene expression analysis. Table S1. Characteristics of the study population: overall cohort and participants with repeated measurements. Figure S1. Manhattan plots of sensitivity analysis. Table S2. Summary of significant CpG sites associated with hypertension persistence. Table S3. Significant associated CpGtranscript pairs. [file 12916_2026_4672_MOESM1_ESM.docx]

**Text S1 Selection criteria of individuals in KORA F4 and FF4**

The KORA F4 study included 3,080 participants, while the KORA FF4 study involved 2,279 participants. Methylation measurements were available for 1,799 participants in KORA F4 and 1,928 in KORA FF4, using the Illumina 450K Infinium Methylation BeadChip and Infinium MethylationEPIC BeadChip, respectively. Samples with greater than 5% missing values (based on the autosomes only) were removed, as well as whose predicted sex differed from the sex recorded at the time of the interview. After quality control, 1,724 individuals remained in KORA F4 and 1,872 in KORA FF4.

The longitudinal analyses of hypertension and blood pressure were restricted to 2,614 participants with at least one DNA methylation measurement at either F4 or FF4. In total, 3,596 observations from 2,614 participants in KORA F4 (1,724) and FF4 (1,872) were included in the analysis. Of these participants, 982 (1,964 observations) had methylation data at both time points.

**Text S2 Methylation Preprocessing Pipeline**

1. DNA methylation measurement：In the KORA F4 study, genome-wide DNA methylation in whole blood was analysed using the Illumina 450K Infinium Methylation BeadChip (Illumina Inc., San Diego, CA, USA). For the KORA FF4 study, the Infinium MethylationEPIC BeadChip (Illumina Inc., San Diego, CA, USA) was used according to standard protocols provided by Illumina. GenomeStudio software version 2011.1 with Methylation Module version 1.9.0 was used for initial quality control of assay performance and for generation of methylation data export files.
2. Reading in the data: Raw IDAT files were read into R (v4.3.0) using the command read.metharray from the Bioconductor package minfi (v1.46.0) and background corrected using the command bgcorrect.illumina.
3. Sex prediction: When we used the command getSex (minfi v1.46.0) on the raw data. In KORA F4, there was no individuals with a predicted sex different from the sex given at the time of the interview (cut-off -1.5). In KORA FF4, there were two individuals with predicted sex different to the sex given at the time of the interview and these individuals were removed.
4. Quality control on raw intensities: We used the command getQC (minfi v1.46.0) on the raw data. In KORA F4, 1 individual failed the QC (cut-off 9) and was removed. In KORA FF4, individuals were removed whose median intensity was less than 50% of the experiment-wide mean, or less than 2,000 arbitrary units (33 individuals).
5. Detection p-value filter: Probes whose detection p-values were greater than 0.01 were set to missing.
6. Sample call rate filter: Samples with greater than 5% missing values (testing the autosomes only) were removed. In KORA F4, this led to the exclusion of 72 individuals. In KORA FF4, 9 individuals were excluded among which 4 individuals were overlapped with those failing raw intensity quality control.
7. CpG call rate filter: In KORA F4, CpG sites with greater than 5% missing values on the autosomes were removed (N= 14,541). In KORA FF4, probes with greater than 5% missing values on the autosomes were also removed (N=5,786).
8. CpG probe exclusion: In KORA F4, we use the manifest HM450.hg19.manifest.pop.tsv.gz (Population-specific masking HM450 file from https://zwdzwd.github.io/InfiniumAnnotation) and set MASK_general_EUR to TRUE to obtain a reliable list of probes to be excluded. This is based on PMID: 27924034. This yields 59,186 CpG sites to exclude. In KORA FF4, 1) Cross-reactive probes: There are publications providing lists for probes that hybridize to multiple possible regions (PMID: 27717381, PMID: 27330998). A total of 44,493 unique probes were removed. 2) SNPs within the probe-binding region: The R package minfi v1.28.3 provides a list of SNPs within the probe-binding regions for each CpG. Probes for CpG sites known to be SNPs with minor allele frequency >0.05 (as given by minfi), or probes that had SNPs in the single base extension with minor allele frequency >0.05 were removed (11,370 and 5,597, respectively).
9. Quantile normalization: Quantile normalization was performed separately on the signal intensities divided into the 6 probe types: type II red, type II green, type I green unmethylated, type I green methylated, type I red unmethylated, type I red methylated (PMID: 25853392). The quantile normalized intensities were then used to generate methylation beta values, a measure from 0 to 1 indicating what percent of the cells were methylated at this locus. This step was performed separately for the autosomes, and for the sex chromosomes. For the sex chromosomes this step was performed separately for men and women. QN was performed using the R package limma v3.56.2 (PMID: 25605792).
10. Blood disorders: In KORA FF4, seven individuals have strong blood disorders. 1 had already been removed due to failing quality control, and the remaining six were removed from the dataset.
11. Cell type heterogeneity: White blood cell type proportions were estimated using the Houseman algorithm (PMID: 22568884) as implemented using the command estimateCellCounts (minfi v1.46.0) on the raw intensities and the default parameters. estimate were performed using the default types: "CD8T", "CD4T", "NK", "Bcell", "Mono",” Gran”.
12. Technical covariates: We calculated the principal components (PCs) of all the non-negative control probes, as per the CPACOR pipeline. Up to 30 control probe PCs can be used as covariates in the regression models to adjust for technical affects. Alternatively, some combination of plate, chip and chip position can be used.
13. Probe count summary: In KORA F4, the original 450K array had 485,577 probes, of which 65 were SNP probes used for quality control and were removed. The array therefore contained 485,512 probes (473,864 on the autosomes, 11,232 on the X chromosome, and 416 on the Y chromosome). A total of 59,186 probes were excluded based on the population-specific masking HM450 file, and 14,541 failed the detection p-value filter, giving 73,727 probes in total. However, some probes overlapped both categories, and the total number removed was 70,640. This left 414,872 probes: 404,837 from the autosomes, 9,792 from the X chromosome, and 243 from the Y chromosome. In KORA FF4, the original EPIC array had 866,895 probes, of which 59 were SNP probes for quality control. A Product Quality Notice (Tracking Number: PQN0223) issued by Illumina on April 19, 2017, indicated that 977 probes were removed due to underperformance, leaving 865,859 probes. Forty samples from batch 1 had defective chips and were missing 598 CpG sites. For these individuals, the missing CpG sites were recorded as missing values in the data. The array therefore contained 865,859 probes (846,232 on the autosomes, 19,090 on the X chromosome, and 537 on the Y chromosome). A total of 44,493 were cross-reactive probes, 11,370 and 5,597 had SNPs in the CG position and single-base extensions respectively, and 5,786 failed the detection p-value filter, giving 67,246 probes in total. Since many probes overlapped multiple categories, 59,631 were removed. This left 806,228 probes: 788,106 from the autosomes, 17,743 from the X chromosome, and 379 from the Y chromosome.
14. Sample count summary: In KORA F4, 1,799 individuals were measured in one batch using the Illumina HumanMethylation 450 BeadChip. A total of 75 were removed due to quality control: these all failed the detection rate threshold, and 1 additionally failed the median intensity step. This leaves 1,724 individuals passing quality control. In KORA FF4, 1,928 individuals were measured in two rounds. 2 were removed due to sex mismatch, 33 removed due to failing quality control on the raw intensities and 9 failed the detection p-value filter (4 overlap with intensity filter), leaving 1888 individuals passing quality control. In the first round, there were N=488 KORA FF4 samples. In the second round, there were N=1,440 KORA FF4 samples. They were both measured using the Illumina EPIC BeadChip. Seven individuals had a noted strong blood disorder or unusual cell counts, one of whom had already been removed from the dataset. The further 6 individuals were removed. After all these steps, 10 individuals withdrew consent for their data to be used, leaving 1,872 individuals.

**Text S3 Selection criteria of CpG sites in KORA F4 and FF4**

Probes with more than 5% missing values on the autosomes were excluded. Additionally, probes containing single nucleotide polymorphisms (SNPs) within the probe-binding regions were removed. Probes were also filtered out if the detection P-value exceeded 0.01, or if they were found to hybridize to multiple genomic regions. Probe intensities were normalized using the quantile normalization procedure for both KORA F4 and FF4. After quality control, 414,872 CpG sites remained in KORA F4 and 806,228 in KORA FF4, with 383,057 overlapping CpG sites. Following the exclusion of sex chromosome CpG sites, 374,054 CpG sites were left in the final analysis.

**Text S4 Quality control for KORA FF4 gene expression data**

After RNA isolation using PAXgene Blood RNA Kit, RNA integrity number (RIN) was measured using the Agilent 2100 Bioanalyzer system. RNA samples with RIN values of approximately 6 or more were selected for mRNA sequencing (poly-A selected). The libraries were prepared using the Illumina stranded mRNA prep ligation kit (Illumina), following the kit's instructions. After a final QC, the libraries were sequenced in a paired-end mode (2x100 bases) in the Novaseq6000 sequencer (Illumina) with a depth of ≥ 40 Million reads per sample. After demultiplexing, FASTQ files from each sample are processed using standard tools. Alignment to UCSC Genome Browser hg19 human reference genome using STAR v2.4.2a (PMID: 23104886). Unaligned reads are discarded. Sequencing QC was done using RNASeQC v1.1.8.1 (PMID: 22539670). Properly aligned reads are then processed with HTSeq-count v0.6.1 (PMID: 25260700) to generate read counts which can be interpreted as quantified gene expression. The reads are then normalized for exon length and total sequencing yield to generate Fragments Per Kilobase of transcript per Million mapped reads (FPKM), and this is done through dividing the fragments per gene by the product of length of the gene in kilobase and million reads sequenced.

After sequencing QC, samples QC was done. Samples with < 30 million reads were discarded. Exonic, intronic, intragenic, intergenic and rRNA rates calculated by RNAseQC were examined for outliers but no such outliers were found, and no samples were excluded based on these. Only the genes with FPKM of ≥ 1 in at least 5% of the samples were selected. Number of the selected genes in each sample were calculated. Samples having less than 5750 genes were excluded. Sex mismatches in the phenotype tables and those discerned from looking at the expression of XIST and UTY genes were also excluded.

**Text S5 DNA methylation and gene expression analysis**

To assess whether the identified CpG sites are associated with gene expression, methQTL analysis was carried out by the MatrixEQTL package. The gene expression data was only available in the KORA FF4 cohort (n = 1,543). Whole-blood gene expression was profiled by short-read RNA sequencing, aligned to the hg19 reference genome via the UCSC Genome Browser, and normalized to fragments per kilobase of feature per million mapped reads (FPKM). Genes with FPKM ≥ 1 in at least 5% of samples were retained, and samples with <5,500 detected genes were excluded. Remaining values were quantile-normalized across samples and genes. DNA methylation β-values at CpG sites were corrected for technical variation by regressing out the first 8 principal components derived from control probes. For transcript counts, first 4 principal components were regressed out. Residuals from both datasets were used in cis-association analyses within a ±500 kb window of each CpG. Linear regression was used to test the associations by adjusting for age, sex, and measured leukocyte subtypes (neutrophils, monocytes, basophils, eosinophils). This combined workflow ensured rigorous quality control, normalization, and adjustment for technical and biological confounders to enable robust detection of local methylation–expression relationships.

Table S1. Characteristics of the study population: overall cohort and participants with repeated measurements.

| **Characteristics** | **All Individuals** | | **Repeated Measurements** | |
| --- | --- | --- | --- | --- |
|  | F4 (N=1724) | FF4 (N=1872) | F4 (N=982) | FF4 (N=982) |
| Age (years) | 61.0 (8.89) | 58.6 (11.6) | 58.4 (8.22) | 64.9 (8.20) |
| Male (%) | 842 (48.8) | 891 (47.6) | 406 (49.3) | 406 (49.3) |
| BMI (kg/m^2^) | 28.1 (4.78) | 27.8 (5.12) | 27.6 (4.56) | 28.0 (4.93) |
| Smoking |  |  |  |  |
| Never smoker (%) | 721 (41.8) | 768 (41.0) | 346 (42.0) | 346 (42.0) |
| Former smoker (%) | 753 (43.7) | 794 (42.4) | 356 (43.2) | 373 (45.3) |
| Current smoker (%) | 248 (14.4) | 310 (16.6) | 122 (14.8) | 105 (12.7) |
| SBP (mmHg) | 125.0 (18.7) | 119.0 (17.2) | 123.0 (17.4) | 121.0 (17.5) |
| DBP (mmHg) | 76.0 (10.0) | 73.4 (9.5) | 76.4 (9.7) | 73.3 (9.5) |
| Diabetes mellitus (%) | 158 (9.16) | 162 (8.65) | 56 (6.80) | 112 (13.6) |
| HDL-cholesterol (mmol/l) | 1.46 (0.38) | 1.69 (0.49) | 1.47 (0.39) | 1.71 (0.49) |
| Triglycerides (mmol/l) | 1.52 (1.01) | 1.40 (0.85) | 1.45 (0.95) | 1.44 (0.82) |
| LDL-cholesterol (mmol/l) | 3.62 (0.91) | 3.49 (0.89) | 3.63 (0.89) | 3.58 (0.91) |
| Cholesterol (mmol/l) | 5.73 (1.01) | 5.60 (1.00) | 5.74 (0.98) | 5.71 (1.03) |

Data are mean (SD) for continuous variables and n (%) for categorical variables.

**
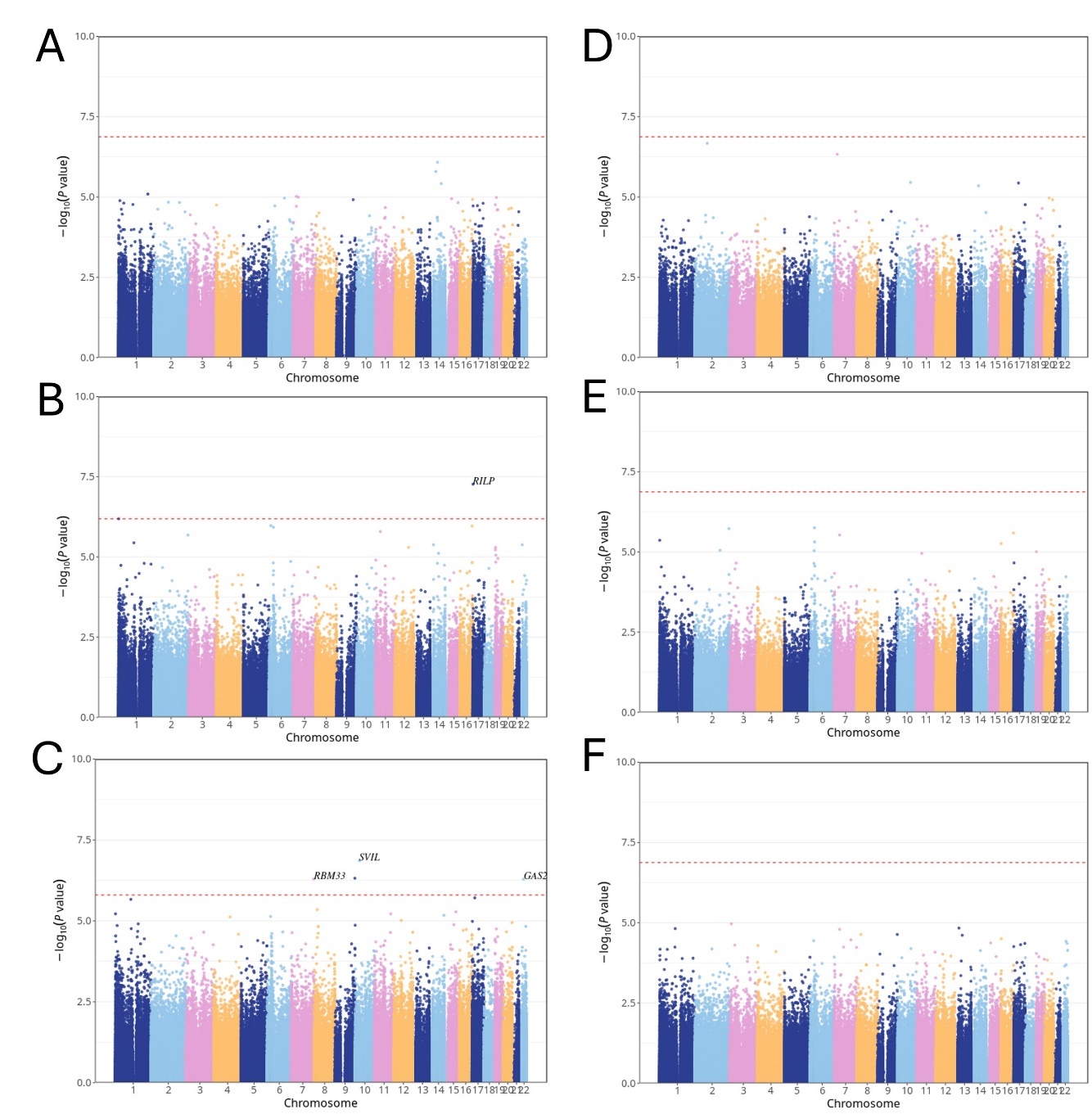
**

**Figure S1**. Manhattan plots of sensitivity analysis. The x axis indicates the chromosome location, and the y-axis represents the −log10 (*p*-value). The Benjamini–Hochberg (FDR) threshold (*p_*FDR < 0.05) is indicated by a red dashed line. (A-C) Manhattan plot from the extended EWAS models for HTN/SBP/DBP, respectively. (D-F) Manhattan plots based on the subset of individuals with two DNA methylation time points for HTN/SBP/DBP, respectively.

**Table S2** Summary of significant CpG sites associated with hypertension persistence.

| **Probe ID** | **BETA** | **P_VAL** | **FDR** | **CHR** | **MAPINFO** | **UCSC_RefGene** |
| --- | --- | --- | --- | --- | --- | --- |
| cg18175690 | 0.03 | 9.61e-10 | 3.59e-04 | 15 | 40580770 | *PLCB2* |
| cg18762620 | -0.01 | 3.71e-09 | 6.93e-04 | 15 | 75940301 | *SNX33* |
| cg18164942 | -0.04 | 2.97e-08 | 3.70e-03 | 14 | 75327747 | *PROX2* |
| cg25033076 | 0.03 | 7.92e-08 | 6.78e-03 | 18 | 11884680 | *MPPE1* |
| cg04319606 | -0.03 | 9.07e-08 | 6.78e-03 | 2 | 26785290 | *C2orf70* |
| cg01819142 | 0.03 | 1.17e-07 | 7.28e-03 | 8 | 139064307 |  |
| cg01564772 | 0.02 | 1.39e-07 | 7.41e-03 | 16 | 8995896 | *USP7* |
| cg10150686 | -0.03 | 1.77e-07 | 8.25e-03 | 2 | 26785332 | *C2orf70* |
| cg01803258 | 0.03 | 3.92e-07 | 1.33e-02 | 1 | 159160407 | *CADM3* |
| cg25633119 | -0.01 | 4.02e-07 | 1.33e-02 | 19 | 48894926 | *KDELR1* |
| cg00610360 | 0.03 | 4.16e-07 | 1.33e-02 | 15 | 99186904 |  |
| cg14382318 | -0.01 | 4.26e-07 | 1.33e-02 | 22 | 46646383 | *C22orf40* |
| cg17176108 | -0.02 | 4.68e-07 | 1.35e-02 | 17 | 40610851 | *ATP6V0A1* |
| cg22734086 | -0.03 | 6.07e-07 | 1.62e-02 | 2 | 26785367 | *C2orf70* |
| cg14086826 | 0.02 | 9.18e-07 | 2.29e-02 | 9 | 138757010 | *CAMSAP1* |
| cg18908524 | -0.01 | 1.31e-06 | 3.06e-02 | 11 | 64002295 | *VEGFB* |
| cg16212881 | 0.00 | 1.70e-06 | 3.59e-02 | 6 | 30640519 | *DHX16* |
| cg17142066 | 0.01 | 1.73e-06 | 3.59e-02 | 8 | 528131 |  |
| cg21340148 | -0.02 | 2.07e-06 | 4.07e-02 | 19 | 2702986 | *GNG7* |
| cg21784274 | 0.02 | 2.39e-06 | 4.47e-02 | 20 | 3645526 | *GFRA4* |

Probe ID: Unique identifier from the Illumina CG database; BETA: The estimate from linear mixed model; FDR: Benjamini-Hochberg corrected *p* value (FDR); CHR: Chromosome; UCSC_RefGene: Target gene name from the UCSC database (# indicates no annotated gene); MAPINFO: Chromosomal coordinates of the CpG (Build 37).

**Table S3** Significant associated CpG-transcript pairs.

| **Probe ID** | **Transcript** | **BETA** | **P_VAL** | **FDR** | **CHR** | **MAPINFO** |
| --- | --- | --- | --- | --- | --- | --- |
| cg03608000 | *gen_ZNF439* | -4.21 | 6.06E-12 | 2.61E-09 | 19 | 11998623 |
| cg25033076 | *gen_TUBB6* | -1.66 | 2.90E-09 | 6.26E-07 | 18 | 11884680 |
| cg25033076 | *gen_MPPE1* | 0.80 | 1.23E-07 | 1.76E-05 | 18 | 11884680 |
| cg03608000 | *gen_ZNF44* | -2.20 | 3.84E-07 | 4.14E-05 | 19 | 11998623 |
| cg00610360 | *gen_IGF1R* | 0.95 | 1.48E-06 | 0.00013 | 15 | 99186904 |
| cg21340148 | *gen_SPPL2B* | -1.38 | 9.18E-06 | 0.00065 | 19 | 2702986 |
| cg25033076 | *gen_AFG3L2* | -0.36 | 1.05E-05 | 0.00065 | 18 | 11884680 |
| cg25033076 | *gen_CHMP1B* | 0.47 | 1.58E-05 | 0.00085 | 18 | 11884680 |
| cg03608000 | *gen_PRKCSH* | -1.40 | 2.56E-05 | 0.00094 | 19 | 11998623 |
| cg03608000 | *gen_ZNF763* | -2.18 | 2.61E-05 | 0.00094 | 19 | 11998623 |
| cg04319606 | *gen_TMEM214* | 0.58 | 2.55E-05 | 0.00094 | 2 | 26785290 |
| cg08625564 | *gen_SLC43A2* | -0.83 | 2.03E-05 | 0.00094 | 17 | 1553453 |
| cg16169375 | *gen_CRIP3* | -1.30 | 4.75E-05 | 0.00158 | 6 | 43197328 |
| cg01803258 | *gen_PYHIN1* | -0.81 | 9.98E-05 | 0.00299 | 1 | 159160407 |
| cg21340148 | *gen_TLE2* | -1.14 | 1.04E-04 | 0.00299 | 19 | 2702986 |
| cg21340148 | *gen_GNG7* | -1.31 | 1.75E-04 | 0.00470 | 19 | 2702986 |
| cg03608000 | *gen_ZNF136* | -1.19 | 2.82E-04 | 0.00715 | 19 | 11998623 |
| cg01065780 | *gen_HMOX1* | -2.89 | 3.73E-04 | 0.00893 | 22 | 36019471 |
| cg01065780 | *gen_MCM5* | -1.43 | 5.37E-04 | 0.01157 | 22 | 36019471 |
| cg12165551 | *gen_EIF3F* | 0.24 | 5.14E-04 | 0.01157 | 11 | 8385712 |
| cg00245850 | *gen_LYPD2* | 1.13 | 0.00131 | 0.02694 | 8 | 143925513 |
| cg24016844 | *gen_WDR77* | -0.80 | 0.00240 | 0.04704 | 1 | 111506641 |

Statistically significant associations between metabolic measure-associated CpG sites and expression of cis-transcripts in whole blood (FDR-adjusted significance threshold *p* < 0.05). BETA: coefficient between methylation and gene transcripts.
